# Image Guidance is Associated with Improved Freedom From Recurrence After Superficial Radiation Therapy for Nonmelanoma Skin Cancer

**DOI:** 10.1016/j.adro.2024.101463

**Published:** 2024-02-09

**Authors:** Erin M. McClure, Geoffrey Sedor, Mairead Moloney, Yuxuan Jin, Lio Yu, Michael W. Kattan

**Affiliations:** a University Hospitals Geauga Medical Center, Chardon, OH; b Columbia University Irving Medical Center, Vagelos College of Physicians & Surgeons, New York, New York; c New York Institute of Technology College of Osteopathic Medicine, Old Westbury, New York; d Department of Quantitative Health Sciences, Cleveland Clinic, Cleveland, Ohio; e Laserderm Dermatology, Smithtown, New York

## Abstract

**Purpose:**

This is the first study to quantify the 2-year freedom from recurrence for individuals with nonmelanoma skin cancer (NMSC) such as basal cell carcinoma (BCC), squamous cell carcinoma (SCC), and squamous carcinoma in situ (SCCIS) treated with image guided superficial radiation therapy (IGSRT) versus SRT without image guidance.

**Methods and Materials:**

This retrospective cohort study evaluates the 2-year freedom recurrence rate of NMSCs treated by IGSRT (March 2016 to January 2022) and compares it to existing data on NMSCs treated by SRT via 1 sample proportion tests. Individuals >18 years old with biopsy-proven SCC, SCCIS, and/or BCC treated with IGSRT were included in the study, and 1602 patients/2880 treated lesions were followed until January 14, 2022. SRT literature was identified through an Ovid Medline search.

**Results:**

All NMSCs treated by IGSRT in this cohort had an aggregate 2-year freedom from recurrence of 99.23%. The recurrence rate for BCC (N = 1382) was 1.1%, for SCC (N = 904) 0.8%, and for SCCIS (N = 594) 0.0%. These recurrence rates are significantly improved compared with a pooled study that evaluated NMSCs across histology and BCCs alone treated without image guidance (standard SRT) (*P* < .001).

**Conclusions:**

IGSRT offers a paradigm-shifting treatment option for patients with NMSCs – offering statistically significantly improved outcomes compared with standard SRT and a more desirable toxicity profile to surgical options. This study demonstrates that IGSRT is associated with remarkably low recurrence rates, which are statistically significantly improved from the previous generation of SRT and in line with modern outcomes for Mohs micrographic surgery.

## Introduction

Nonmelanoma skin cancers (NMSCs) are primarily comprised of basal cell carcinomas (BCC) and squamous cell carcinomas (SCC).^1^ Although they are not nearly as fatal as melanoma, they can be a source of significant morbidity, and they are the most common malignancy found in the United States.^2^ Unfortunately, their incidence is rising in many areas of the world, including the United States,^2^, ^3^, ^4^ where incidence is increasing by approximately 2% annually.^5^ The incidence of NMSCs in the United States was estimated to be 5.4 million cases in 2012, which is a 35% increase since 2006.^6^

Currently, the primary treatment modality for low-risk NMSCs is surgical resection.^1^ Patients with advanced disease are often treated with systemic therapies and in clinical trials or with comprehensive radiation therapy (RT). For patients with localized disease but who are poor surgical candidates (comorbidities/advanced age, inoperable location, morbid surgical outcomes, etc), definitive RT is preferred. Definitive dosing tends to range from 30 to 60 Gy.^7^ RT can also be useful as a postoperative approach, especially when there are close or positive surgical margins. Additionally, it can act as a palliative option in the setting of advanced disease.^8^

Superficial RT (SRT) is a type of external RT that uses a lower level of energy, which limits penetration to tissues beyond the skin. Dosing schedules for SRT typically vary from 5 Gy/fraction x 7 fractions (35 Gy total) to 2 Gy/fraction x 30 fractions (60 Gy total) or more depending on age of the patient, lesion location, and lesion size.^9^ This modality has been in use for over a century and was commonly practiced in the 1970s, with up to 55% of dermatology clinics using SRT in 1974.^7^ SRT was phased out as the preferred modality secondary to the advent of Mohs micrographic surgery (MMS), which has a superior recurrence-free survival to SRT (MMS has 99% 5-year recurrence-free survival for BCC and 97% for SCC vs SRT, which has 96% 5-year recurrence-free survival for BCC and 94% for SCC).^10^^,^^11^

Image guided superficial RT (IGSRT) employs the use of ultrasound technology to better visualize the cancerous lesion, which allows for more precise targeting of the radiation by accurately assessing the tumor margin and determining the necessary depth of treatment. Specifically, an ultrasound set to a frequency of 22 MHz (optimal for assessing the skin layer at a depth of 0-6 mm) is used to determine the extent of the lesion beyond what is clinically visible.^12^ We hypothesize that the improved NMSC visualization because of the use of ultrasound to guide SRT will be associated with improved the 2-year freedom from recurrence rates of these lesions. Data supporting this hypothesis would help shift the paradigm of RT for the use of NMSCs and help make it a more viable option for certain patients who are poor candidates for MMS or for whom the toxicity profile is more favorable.

## Methods and Materials

The ethics committee/institutional review board waived ethical approval for this work. Treatments with IGSRT were initiated between March 28, 2016, and January 6, 2020. The initial data set was obtained from a published article that included 2917 lesions; however, 19 lesions had no follow-up after treatment completion, 14 lesions had mixed histologies, and 4 lesions had no histology recorded, so these lesions were excluded.^12^ Patients were followed until January 14, 2022, by which time 22 lesions had recurred. Using an 18-month cutoff for recency of assessment, 1204 lesions (41.8%) were lost to follow-up (ie, had not been assessed after July 14, 2020). The median follow-up among patients who remain alive and did not recur was 26.3 months (1st and 3rd quartiles, 10.0 and 38.4 months, respectively), with a maximum of 50.1 months.

Time to lesion recurrence was estimated using the cumulative incidence method with death of the host as a competing risk. Observed 2-year probabilities were compared with those of the literature with a test of proportions.

### SRT literature search

A search of the Ovid Medline database was conducted with the following strategy:

1. nonmelanoma skin cancer.mp.
2. basal cell carcinoma.mp. or exp Carcinoma, Basal Cell/
3. squamous cell carcinoma.mp. or exp Carcinoma, Squamous Cell/
4. squamous carcinoma in situ.mp. or exp Carcinoma in Situ/
5. 1 or 2 or 3 or 4
6. exp Recurrence/ or exp Neoplasm Recurrence, Local/ or recurrence.mp.
7. image guided superficial radiation therapy.mp. or exp Radiation therapy, Image Guided/
8. ((conventional or superficial) adj3 radiation therapy).mp.
9. 7 or 8
10. 5 and 6 and 9

**Figure 1:**
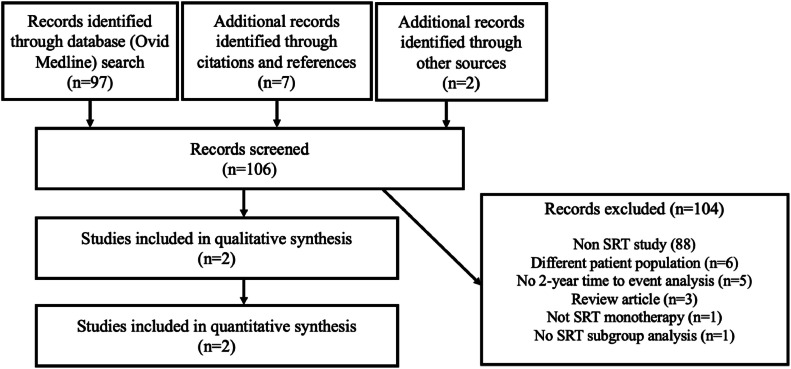
Flow diagram of superficial radiation therapy article selection.

This search identified 97 articles (see Table E1). A thorough review of each article's references and citations revealed 5 additional potentially relevant papers. Articles not focused on SRT, animal studies, those with no 2-year recurrence data, and reviews were excluded. Five articles suitable for comparison remained (see Fig. 1).

### Detailed IGSRT treatment methodology and energy/dose selection process

On the day of the simulation, the lesion(s) is examined including measurements of surface dimensions such as length, width, and maximal diameter. Maximal lesion depth is measured using a 22-MHz high resolution dermal (HRD) ultrasound (US) probe. A minimum radial treatment margin of 5 mm for small superficial lesions (<2 cm in max diameter and <1-mm thickness) is generally used for stage I lesions, and 5- to 10-mm (or greater) treatment margin is used for ≥stage II, thicker lesions (≥1-mm thickness), or those with more aggressive subhistologies (nodular, micronodular, morpheaform, infiltrative, etc, BCCs and most cutaneous SCCs). Margins are marked on the skin surface and photographed with setup instructions documented. Final margin determination is subjected to the discretion of the provider/radiation oncologist. Custom shielding with a lead shield cutout with a minimum 0.762-mm thickness is used unless the selected circular aperture of the treatment cone corresponds well to and entirely covers the lesion with adequate margins. Margin modifications because of nearby critical structures or other clinical factors may be performed at the direction of the provider/radiation oncologist. Treatment energy selected initially and throughout treatment is determined by the deepest measurement of the lesion on the HRD US at initial simulation and before each treatment with energy guidelines based on percentage depth dose data available from the manufacturer (Sensus Healthcare) and is “adaptive.” This adaptive method can facilitate potentially better local control and/or fewer possible complications by adapting to tumor growth/regression/bleeding or tissue edema or other factors such as trauma and so forth. Energy selection of 50, 70, or 100 kV is determined and approved by the provider/radiation oncologist.

Although there are many potential fractionation schemes for treatment of NMSC ranging from standard daily fractionation of 180 to 200 cGy 5 times a week to markedly hypofractionated regimens (at or above 500 cGy), the vast majority of the lesions were located in cosmetically sensitive areas (head and neck) where lower dose per fraction was advisable for best cosmesis (which is consistent with current National Comprehensive Cancer Network guidelines). However, most lesions are sufficiently small that a moderately hypofractionated approach delivering 250 to 300 cGy daily fraction 3 to 4 times a week is generally favored. A treatment protocol guideline developed in 2017 and refined in 2019 recommends energy, daily dose ranges, and biologic dose ranges using a time-dose-fractionation table/calculation. This guideline is generally adhered to in this patient population and was previously published.^12^

### Tumor configuration and depth determination

As the cutaneous lesion depth is particularly difficult to assess clinically by physical examination, the maximal tumor depth and size determination is facilitated and greatly improved by the use of the high-resolution dermal US. This also provides the clinician a basis for energy selection. On visualization by the HRD US, the tumor is generally seen as a uniformly hypoechoic “dark” region, often with distinct borders emanating from the underside of the epidermis (which appears as a thin, yellow hyperechoic stripe) expanding down into the underlying normal dermis (which appears as a thick, green hyperechoic region). This depth of involvement measurement supplements and increases the accuracy of direct palpation by the provider.

### Pooled analysis method

In addition to individual comparisons by histology of reported groups, we desired to compute an overall, pooled outcome comparison. To conduct this comparison of the IGSRT cohort to those reported in the literature, we pooled results from 2 reference groups that reported enough granularity of outcome to compute 2-year freedom from recurrence rates. To do so, we calculated the weighted average of the 2-year freedom from recurrence rates for patients from the 2 reference groups for comparison to ours. The weights were obtained as the sample size of each reference study relative to the total sample size combining the 2 studies. A similar approach was used to determine recurrence rate for all patients in the reference groups with BCC (which was included in both published groups’ cohorts). The IGSRT recurrence probabilities and these pooled estimates were compared using a test of proportions. Pooling individual subject data enhances statistical power, improving the ability to compare outcomes across treatments.

## Results

### Patient and tumor characteristics

From the non-IGSRT studies, we reviewed the published tables, figures, and texts and found comparable patient and tumor characteristics to the IGSRT cohort to allow meaningful comparison with regard to lesion sizes, stages, anatomic locations, ages, sexes, and histologies.

The median age of patients at their first treatment with IGSRT was 74 (1st and 3rd quartiles 67 and 80; see Table 1 for further details). In the Cognetta et al study patient/lesion characteristics showed predominance of males at 2:1 ratio with median age at diagnosis of 79 years old.^11^ The locations of the lesions also showed similar preponderance in the head and neck region (the reader is referred to their Table 1 for details as well as the Table 1 in this paper). The Silverman cohort also showed a preponderance of head and neck lesions as described in their first 840 BCC lesions.^13^ The majority of NMSCs treated by IGSRT were BCCs at 48.0% (N = 1382), with SCCs comprising 31.4% (N = 904), and 20.6% (N = 594) were squamous carcinoma in situ (SCCIS). BCCs were also the most likely to recur, with a 2-year recurrence rate of 1.1% versus 0.8% for SCC and 0.0% for SCCIS. Most NMSCs were found on the head/neck (66%), where cosmesis becomes ever more important. The vast majority of NMSCs were stage I (66%) and stage 0 for SCCIS (14%). The full clinical details can be found in Tables E2 and E3 describing stage and event type for each histology in our study (our cohort included NMSC in many anatomic locations; for details see Table E2. A subset analysis including site is planned in future work). The initial tumor size distribution of the IGSRT cohort can be found in Table 2 of the 2021 Yu et al^12^ paper indicating only stage Tis, T1, and T2 lesions with a mean lesion size of 1 cm. In the Silverman et al^13^ publication, there are limited data regarding the exact patient/lesion characteristics in their part 1 publication. However, the Silverman treatment methodology followed a previously published New York University (NYU) standardized protocol showing similar early stage BCCs. The lesions in the NYU cohort were divided into roughly equal groups of 2 to 7 mm, 8 to 10 mm, and 11 to 15 mm. There was also a smaller group of lesions >16 mm (latter group actually showing a higher local control rate than the other smaller lesion groups).^14^ Thus, the IGSRT size distribution is similar to that reported in Silverman et al (median size previously reported to be 10 mm) and matches the same staging categories as in Cognetta et al Table 3, which indicates Tis, T1, and T2 lesions only.

**Table 1:** Cohort characteristics

Characteristic	
Lesions	2880
Patients	1602
Age*	74 (67, 80)
Sex	
Male	913 (57.0%)
Female	689 (43.0%)
Histology	
BCC	1382 (48.0%)
SCC	904 (31.4%)
SCCIS	594 (20.6%)
Stage	
0	594 (21%)
1	1896 (66%)
2	390 (14%)
Follow up (months)*	26.3 (10.0, 38.4)
Event	
Death (other cause)	70
Recurrence	22
Total dose (Gy)†	51.9 (37.2, 73.6)
Total # of fractions†	20.0 (20, 30)
Dose per fraction (Gy) †	2.59 (1.84, 3.83)

*Abbreviations:* BCC = basal cell carcinoma; SCC = squamous cell carcinoma; SCCIS = squamous carcinoma in situ.

⁎ Median (IQR); n (%).

† Median (min, max).

Of the 2880 lesions in the IGSRT cohort, 833 lesions recorded use of multiple energies, revealing approximately a 29% change in the energy prescription (see Table E4).

Adverse events have been reported previously,^12^ but overall, this treatment is exceptionally well tolerated. In brief, using the Radiation Therapy Oncology Group (RTOG) skin toxicity grading system, of the 2154 lesions with documented RTOG grading, 79% (n = 1698) were grade 1, 20% (n = 436) were grade 2, 0.7% were grade 3, and 0.2% were grade 4.

### Outcomes

**Figure 2:**
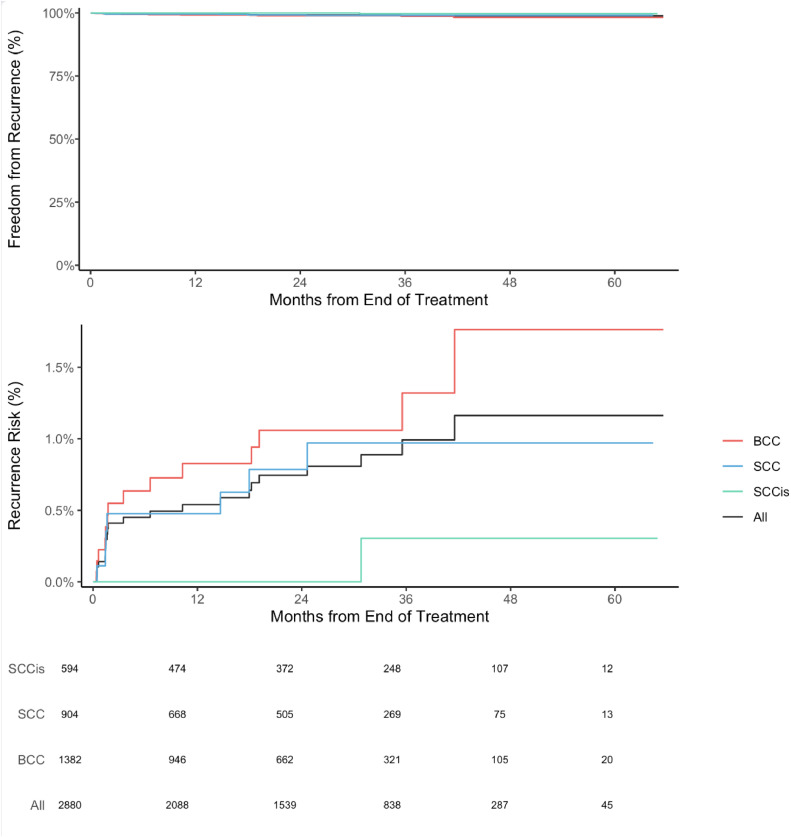
Freedom from recurrence over time of nonmelanoma skin cancers treated with image guided superficial radiation therapy. Top: Freedom from recurrence for all evaluated nonmelanoma skin cancers (squamous cell carcinoma, squamous carcinoma in situ, basal cell carcinoma). Middle: Cumulative incidence of recurrence for squamous cell carcinoma (light blue), squamous carcinoma in situ (blue), basal cell carcinoma (red), and all histologies (black). Bottom: Number of evaluable patients over time.

In this study's cohort of 2880 lesions of 1602 patients treated primarily with IGSRT, 22 IGSRT patients experienced a recurrence of their NMSC and 70 patients died of causes unrelated to their NMSC. The 2-year recurrence rate of overall NMSC lesion recurrence was 0.7% (N = 2880; see Fig. 2, top). When separated by histology, BCC had a 2-year recurrence rate of 1.1% (N = 1382), SCC was 0.8% (N = 904), and SCCIS was 0.0% (N = 594; Fig. 2, middle). This contrasts with the Cognetta et al study, which evaluated 1715 lesions treated by SRT and had an overall 2-year NMSC recurrence rate of 1.9%, more than double that of the cohort studied here.^11^ By histology, Cognetta et al reported 2.0% for BCCs, 1.8% for SCCs, and 1.9% for SCCIS. The Silverman et al study, which used SRT for 3900 BCCs, had a 2-year recurrence rate of 6.3%.^13^

### Comparison of recurrence rates

In the primary analysis, we compared recurrence data from all histologies in our IGSRT cohort directly to pooled data from the Cognetta et al and Silverman et al SRT studies together. Comparing these 2 pooled groups’ 2-year freedom from recurrence revealed a significantly improved outcome for the patients treated with IGSRT versus SRT (0.7% vs 5.8%, respectively; *P* < .001).

**Figure 3:**
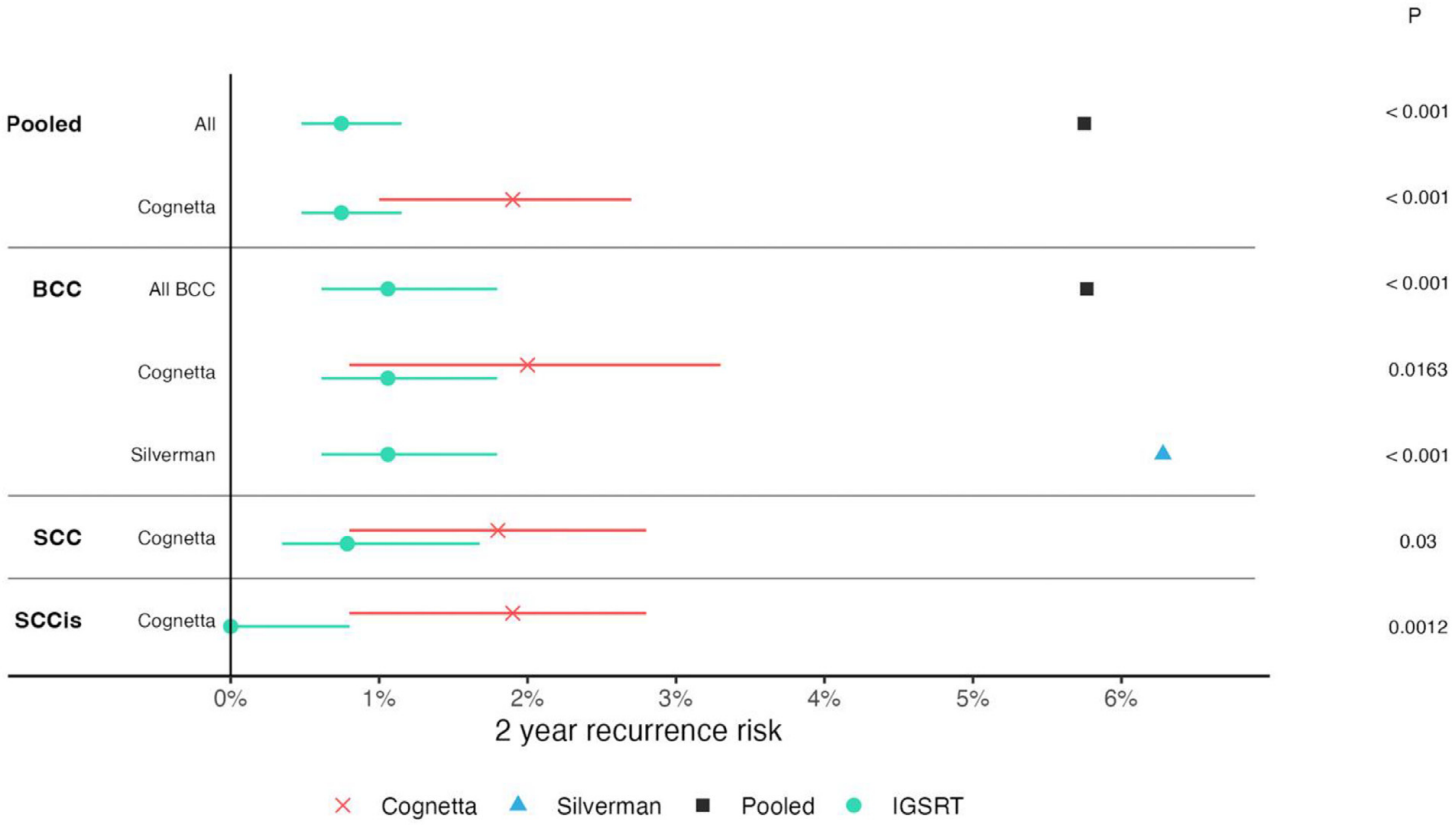
Forest plot of nonmelanoma skin cancer 2-year freedom from recurrence, image guided superficial radiation therapy versus superficial radiation therapy. Red Xs represent data from Cognetta et al^11^ study and blue triangle data from Silverman et al.^13^ Black squares represent pooled superficial radiation therapy cohorts for comparison. Circles represent median recurrence rate for our image guided superficial radiation therapy cohort, and lines represent 95% CIs. Those intervals were not reported by Silverman and are not possible to compute for the pooled estimates without access to patient-level data.

As a secondary analysis, we compared the 2 traditional SRT studies and our own data, subdivided by histology. All secondary histologic head-to-head comparisons show statistically significant differences favoring IGSRT: SCCIS (*P* = .001), BCC (*P* = .016), and SCC (*P* = .03) lesions as reported by Cognetta et al and BCC as reported Silverman et al (*P* < .001; see Fig. 3).

## Discussion

### Major findings

The major findings of this study are the statistically significantly improved 2-year recurrence rates of NMSC when treated primarily with IGSRT compared with SRT. This held true for every comparison made between IGSRT and SRT (pooled and stratified by histology). This improved recurrence rate with IGSRT indicates that radiation may be a viable therapeutic option for patients with early-stage NMSCs, like BCC, SCC, and SCCIS, or those who cannot tolerate or are ill-suited for surgical resection.

### Adverse reactions

IGSRT is remarkably well tolerated by patients. Greater than 99% of adverse events were only RTOG grade 1 or 2 cutaneous toxicities (erythema, epilation, patchy desquamation, moderate edema, and/or decreased sweating). All toxicities recovered fully, most within 2 weeks. Details on the adverse reaction in this cohort have been reported previously.^12^

### Additional SRT studies

Notable mentions from the SRT literature search include Barysch et al,^15^ Zagrodnik et al,^16^ Piccinno et al,^17^ and Caccialanza et al.^18^ All these studies reported recurrence data for BCCs and/or SCCs treated with SRT. Barysch et al^15^ was excluded from this analysis because it included 3 relapsed SCCs in its data, which are a higher grade than the comparison IGSRT group. The 2-year recurrence rate of the 180 reported SCCs was 8.5%, but this number could be higher than the IGSRT group because of these higher risk lesions. Zagrodnik et al^16^ did not explicitly state a 2-year recurrence rate and was therefore excluded from this analysis. However, according to Table 4, the recurrence rate of their 47 NMSCs was approximately 7%. Similarly, Piccinno et al^17^ was excluded for lack of explicit 2-year recurrence data. Per Table 3, 4% to 6% of BCCs recurred between 12 to 24 months, depending on their histologic subtype, with a cumulative sample size of 132 at this time interval. Caccialanza et al^18^ tracked the recurrence of 671 NMSCs treated with SRT. However, it was excluded for no 2-year event analysis, and the inclusion of 89 high risk, relapsed lesions. Based on Figure 1, the 2-year recurrence rate of this cohort was approximately 6%.

### Limitations

A limitation of this study is its retrospective design, as randomized controlled trials are the gold standard for treatment comparisons. Therefore, correlations can be assessed, but causation cannot be. Additionally, not enough time has elapsed yet to determine 5-year recurrence rates, which are a common endpoint in evaluating recurrences of NMSCs.

### Summary

In summary, the 2-year freedom from recurrence for individuals with NMSC treated with IGSRT was statistically significantly improved from those without image guidance and is on par with that of modern surgical technique. This implies that IGSRT may be an effective and valuable treatment option for BCCs, SCCs, and SCCIS when patients are not good candidates for surgical removal or when they refuse surgery and decide the toxicity profile of IGSRT is more desirable. Evaluating the risks of recurrence based on specific patient and tumor characteristics such as age, patient ethnicity, tumor size, tumor site, and eventually genomics, will help better characterize the patient populations that would receive the most benefit from IGSRT.^19^

Patients experience excellent outcomes when their early-stage NMSCs are treated by IGSRT. Specifically, minimal side effects and high satisfaction scores are associated with the standard dosing regimen of IGSRT.^20^ Additionally, excellent 2-year freedom from recurrence rates result from NMSCs treated by IGSRT. Taken together, these data suggest that IGSRT may be a critical technological step forward in providing modern, MMS-scale local control of NMSCs with a very tolerable toxicity profile.

## Conclusion

To our knowledge, this cohort study is the first to directly evaluate and compare 2-year freedom from recurrence rates in NMSC lesions primarily treated with IGSRT versus SRT considering competing risks. We reported 2-year recurrence data for BCCs, SCCs, and SCCIS, both separately and overall, for lesions treated primarily by IGSRT and then compared this to existing data on the recurrence rates for these cancers treated by SRT. We found that overall, NMSCs that received IGSRT had a 2-year recurrence rate of 0.7%, which, compared with the pooled SRT recurrence rate of 5.8%, was statistically significantly improved (*P* < .001). Although no head-to-head trial data exist, this recurrence rate is on par with MMS and signifies that IGSRT is an excellent option for patients who cannot undergo surgical removal of their NMSCs or who refuse surgery and instead desire a different toxicity profile.

## Disclosures

Lio Yu and Michael W. Kattan are paid consultants for SkinCure Oncology. No other authors have conflicts of interest to declare.

## Appendix Supplementary materials

Supp Table 1 **Supplemental Table 1.** Spreadsheet of all articles identified through Ovid Medline search. Image, application 1

Supp Tables 2 **Supplemental Table 2.** Tumor sites included in our cohort of patients treated with IGSRT, stratified by histology. Staging and events by histology. **Supplemental Table 3.** Clinical characteristics stratified by histology. **Supplemental Table 4.** Number of lesions separated by energy of treatment. Image, application 2

## Footnotes

Supplementary material associated with this article can be found in the online version at doi:10.1016/j.adro.2024.101463.
