# Supplementary material for: Image Guidance is Associated with Improved Freedom From Recurrence After Superficial Radiation Therapy for Nonmelanoma Skin Cancer
Source: Adv Radiat Oncol. 2024 Feb 9;9(12):101463. doi: 10.1016/j.adro.2024.101463 (PMC11704900; doi:10.1016/j.adro.2024.101463)
Supplement: Supp Tables 2 — Supplemental Table 2. Tumor sites included in our cohort of patients treated with IGSRT, stratified by histology. Staging and events by histology. Supplemental Table 3. Clinical characteristics stratified by histology. Supplemental Table 4. Number of lesions separated by energy of treatment. [file mmc2.docx]

**Supplemental Table 2.** Tumor sites included in our cohort of patients treated with IGSRT, stratified by histology. Staging and events by histology. Percent of lesions in each site by histology type included.

| **Site** | **BCC, N = 1,382** | **SCC, N = 904** | **SCCis, N = 594** | **Total, N = 2,880** |
| --- | --- | --- | --- | --- |
| Head and Neck (H&N) | 1,003 (73%) | 537 (59%) | 371 (62%) | 1911 (66%) |
| *H&N sublocation* | | | | |
| Ear | 121 | 95 | 54 | 270 |
| Scalp | 52 | 83 | 58 | 193 |
| Forehead | 104 | 73 | 74 | 251 |
| Temple | 37 | 19 | 12 | 68 |
| Forehead/Temple | 0 | 0 | 1 | 1 |
| Eyebrow | 5 | 2 | 4 | 11 |
| Eyelid | 24 | 2 | 1 | 27 |
| Nose | 350 | 78 | 46 | 474 |
| Cheek | 191 | 120 | 92 | 403 |
| Cutaneous Lip | 48 | 13 | 7 | 68 |
| Mucosal Lip | 3 | 13 | 4 | 20 |
| Chin | 6 | 0 | 0 | 6 |
| Jawline | 2 | 3 | 0 | 5 |
| Chin/Jawline | 1 | 0 | 0 | 1 |
| Neck | 59 | 35 | 18 | 112 |
| Other | 0 | 1 | 0 | 1 |
| Extremities | 224 (16%) | 319 (35%) | 191 (32%) | 734 (25%) |
| *Extremities sublocation* | | | | |
| Hand | 11 | 78 | 53 | 142 |
| Other | 153 | 226 | 128 | 507 |
| Shoulder | 60 | 15 | 10 | 85 |
| Trunk | 152 (11%) | 47 (5%) | 31 (5%) | 230 (8%) |
| *Trunk sublocation* | | | | |
| Chest | 45 | 26 | 10 | 81 |
| Back | 96 | 20 | 21 | 137 |
| Other | 11 | 0 | 0 | 11 |
| Penis | 0 | 1 | 0 | 1 |
| Not recorded* | 3 | 1 | 1 | 5 |

**Supplemental Table 3.** Clinical characteristics stratified by histology.

| **Characteristic** | **BCC, N = 1,382** | **SCC, N = 904** | **SCCis, N = 594** |
| --- | --- | --- | --- |
| Stage | | | |
| 0 | 0 | 0 | 594 |
| 1 | 1,150 | 746 | 0 |
| 2 | 232 | 158 | 0 |
| Event | | | |
| Death (other cause) | 30 | 29 | 11 |
| Recurrence | 14 | 7 | 1 |
| Follow Up (months)* | 23.3 (8.3, 35.8) | 28.3 (11.4, 38.4) | 32.9 (15.1, 43.1) |

N; *Median (IQR)

**Supplemental Table 4.** Number of lesions separated by energy of treatment.

| **Energy (kV)** | **# of lesions** |
| --- | --- |
| 50 | 1372 |
| 70 | 632 |
| 100 | 34 |
| 50/70 | 753 |
| 50/100 | 0 |
| 70/100 | 70 |
| 50/70/100 | 10 |
| No Energy Data | 9 |
| TOTAL Lesions | 2880 |
